# Posttraumatic stress disorder symptoms and television viewing patterns in the Nurses’ Health Study II: A longitudinal analysis

**DOI:** 10.1371/journal.pone.0213441

**Published:** 2019-03-21

**Authors:** Sun Jae Jung, Ashley Winning, Andrea L. Roberts, Kristen Nishimi, Qixuan Chen, Paola Gilsanz, Jennifer A. Sumner, Cristina A. Fernandez, Eric B. Rimm, Laura D. Kubzansky, Karestan C. Koenen

**Affiliations:** 1 Department of Epidemiology, Harvard T.H. Chan School of Public Health, Boston, Massachusetts, United States of America; 2 Department of Preventive Medicine, Yonsei University College of Medicine, Seoul, Korea; 3 Department of Social and Behavioral Sciences, Harvard T.H. Chan School of Public Health, Boston, Massachusetts, United States of America; 4 Department of Environmental Health, Harvard T.H. Chan School of Public Health, Boston, Massachusetts, United States of America; 5 Department of Biostatistics, Mailman School of Public Health, Columbia University, New York, New York, United States of America; 6 Kaiser Permanente Division of Research, Oakland, California, United States of America; 7 Center for Behavioral Cardiovascular Health, Columbia University Medical Center, New York, New York, United States of America; 8 Channing Division of Network Medicine, Department of Medicine, Brigham and Women's Hospital, Boston, Massachusetts, United States of America; Stellenbosch University, SOUTH AFRICA

## Abstract

**Introduction:**

The relation between TV viewing and posttraumatic stress disorder (PTSD) is controversial; prior work focused exclusively on whether TV viewing of disaster events constitutes a traumatic stressor that causes PTSD. This study evaluates a possible bidirectional relation between PTSD and TV viewing in community-dwelling women.

**Methods:**

Data are from the PTSD subsample of the Nurses’ Health II study, an ongoing prospective study of women aged 24–42 years at enrollment and who have been followed biennially (N = 50,020). Trauma exposure and PTSD symptoms (including date of onset) were assessed via the Brief Trauma Questionnaire and the Short Screening Scale for DSM-IV PTSD. Average TV viewing was reported at 5 times over 18 years of follow-up. Linear mixed models assessed differences in TV viewing patterns by trauma/PTSD status. Among women with trauma/PTSD onset during follow-up (N = 14,374), linear spline mixed models assessed differences in TV viewing patterns before and after PTSD onset.

**Results:**

Women with high PTSD symptoms reported more TV viewing (hours/wk) compared to trauma-unexposed women at all follow-up assessments (β = 0.14, SE = 0.01, p < .001). Among the women who experienced trauma during follow-up, significant increases in TV viewing (hours/day) prior to onset of high PTSD symptom levels were evident (β = 0.15, SE = 0.02, p < .001).

**Conclusions:**

TV viewing following trauma exposure may be a marker of vulnerability for developing PTSD and also a consequence of having PTSD. High TV viewing levels may be linked with ineffective coping strategies or social isolation, which increase risk of developing PTSD.

## Introduction

The relation between TV viewing and posttraumatic stress disorder (PTSD) has been highly controversial. Following the September 11^th^ terrorist attacks, much literature focused on whether repetitive viewing of disasters on TV constitutes a traumatic stressor that could cause PTSD, with cross-sectional studies reporting positive associations and longitudinal studies reporting mixed findings [1–6]. In 2007, three longitudinal studies[1, 4, 6] assessed the impact of TV viewing on PTSD development, and only one study[1] succeeded in finding a significant result. A study[5] in 2011 among soldiers in the UK did not show any significant result between TV watching and PTSD symptoms. However, in 2012, a longitudinal study[2] of 141 US citizens who were indirectly exposed to both hurricanes Katrina and Gustav through TV viewing reported an increased level of PTSD symptoms. Similar results observed in a longitudinal study of response to Oklahoma City bombing in 2016[3]. Ultimately, the Diagnostic and Statistical Manual of Mental Disorders (DSM)-5 specifically excluded witnessing traumatic events on TV as a traumatic stressor [7].

However, this narrow focus precluded a broader investigation into the relation between TV viewing and PTSD. Whether TV viewing might serve as a risk marker of PTSD or, conversely, whether PTSD onset is associated with greater subsequent TV viewing has not been established. Excessive TV viewing may be a marker of social isolation or poor coping [8], which in turn increases the risk of developing PTSD following exposure to trauma. TV viewing has been associated with a range of mental health problems including depression [9], antisocial behavior, and ADHD [10]. Thus, TV viewing may indicate vulnerability to PTSD. However, PTSD is associated with increases in other adverse health behaviors, and for similar reasons PTSD may be prospectively associated with increased TV viewing. For example, women engage in less physical activity and more smoking after PTSD onset compared with trauma-exposed women who do not develop PTSD [11, 12]. Given PTSD symptoms of avoidance have been linked to increased social isolation, PTSD may lead to more TV viewing due to less time spent in other activities [8].

To our knowledge, only one longitudinal study has examined whether exposure to trauma subsequently changed TV viewing hours [13]. In a study of women and children who experienced the September 11^th^ terrorist attacks indirectly via media sources, the majority of respondents (64%) reported watching “a lot more” TV following the attacks. However, PTSD symptoms were not assessed in this study.

Here we evaluate a possible bidirectional association between TV viewing and trauma/PTSD symptoms over 18 years in the Nurses’ Health Study II (NHS II), a cohort of women enrolled in 1989. We examined whether more TV viewing in general (e.g. not associated with a specific event) is associated with subsequently increased risk of PTSD. Additionally, we examine whether PTSD onset was associated with increases in TV viewing. We consider the role of trauma alone versus PTSD and further adjust for history of depression symptoms, as depression is commonly comorbid with PTSD [14, 15].

## Materials and methods

### Study population

**Fig 1:**
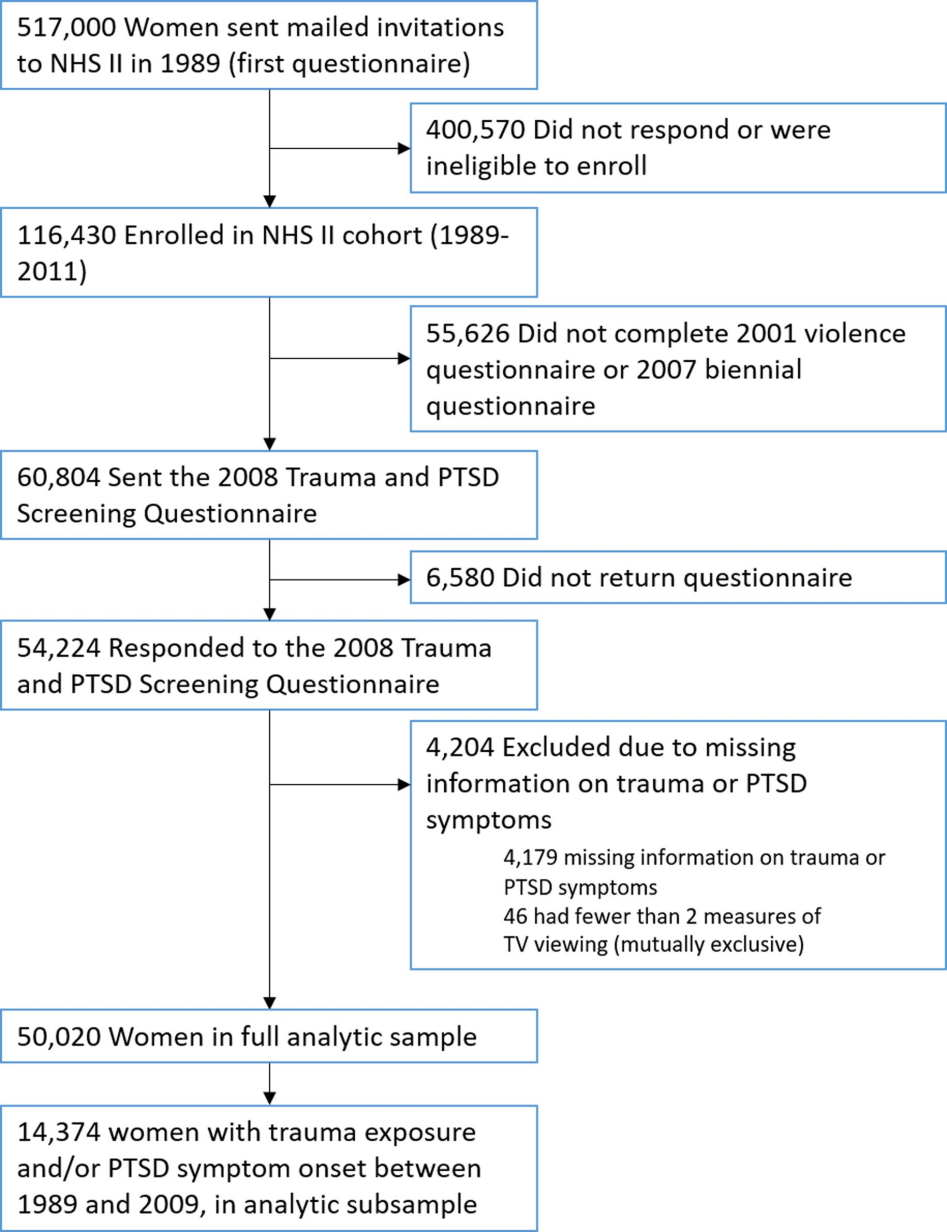
Flowchart of exclusions for deriving the analytic samples.

The NHS II consists of 116,429 US registered nurses, aged 24–42 years at enrollment in 1989 and followed biennially. Women were predominantly white (N = 106,161, 91.0%) and relatively highly educated, having received at least a two-year nursing degree, residing 14 states which were the regions from the northeast, the mid-west, the southern and the west of USA. In 2008, a supplemental questionnaire querying trauma and PTSD [16] was mailed to 60,804 participants who had completed an earlier violence questionnaire. A total of 54,224 women returned the questionnaire (89% response rate). Women missing trauma/PTSD data or with fewer than two measures of TV viewing were excluded (n = 4,204), leaving 50,020 respondents for the analyses in 2017 (Fig 1). Women included in the main analyses showed similar baseline characteristics with women who were excluded. A subsample of 14,374 women reported that their worst trauma occurred during follow-up, between 1989 and 2009 (Fig 1). This study was approved by Partners Healthcare Internal Review Board.

### Measures

#### Trauma & PTSD

A modified version of the Brief Trauma Questionnaire [17, 18] was used to assess lifetime exposure to any 16 potentially traumatic events (e.g., serious accident, physical assault). Presence and frequency of PTSD symptoms related to participants' worst identified trauma, including avoidance and mood swing where assessed using the 7-item Short Screening Scale for DSM-IV PTSD [16]. Responses indicating the frequency of each PTSD symptom in the past 4 weeks were rated on a scale from 0 (none of the time) to 4 (most of the time), with a possible total score of the summed item responses ranging from 0 to 28. The cutoff of 4 on this scale showed about 80% of sensitivity, 97% of specificity, 71% of positive predictive value, and 98% of negative predicted value, and it appeared to be the best overall cutoff point by the developers of the screening scale for DSM-IV PTSD; this was shown to be an efficient instrument to screen for PTSD in epidemiologic settings [16]. Onset of PTSD was established according to age reported at worst trauma, using a specially designed question sequence developed to improve accuracy of age of onset of reporting of psychiatric disorders [19]. Reliability of self-reported age of onset of trauma and PTSD has been found to be excellent in this sample (intraclass correlation, 0.95) [20].

Trauma and PTSD were coded as time varying. For each year of the study, participants were categorized into 1 of 5 groups based on the year at which the *worst* traumatic event occurred: (1) no trauma exposure, (2) trauma exposure but no PTSD symptoms, (3) trauma exposure with 1–3 PTSD symptoms, (4) trauma exposure with 4–5 PTSD symptoms, and (5) trauma exposure with 6–7 PTSD symptoms [16, 21]. For example, if a woman reported that her only and worst trauma occurred in 1993, and reported four symptoms subsequent to that trauma, she was classified as having no trauma exposure from 1989 through 1992, and as having trauma and four PTSD symptoms from 1993 through the end of follow-up. Women were considered as having no trauma exposure in either case of having no trauma or before the first trauma, and after their first trauma, women were coded as being trauma-exposed with no symptoms.

#### Television viewing

TV viewing was assessed in 1991, 1997, 2001, 2005, and 2009 with the question: “During the past year, on average, how many hours per week did you spend sitting at home while watching TV/VCR/DVD?” To reduce the effect of outliers, responses were Winsorized at 90 hours/week (99^th^%ile). In a validation study, responses to this question had moderate correlation with 24-hour TV viewing recall (r = 0.54) [22].

#### Covariates

Covariates were selected based on their relation to both PTSD and health behaviors in prior work [23–26]. In 1989, participants reported their age, race/ethnicity and childhood adiposity. To assess childhood adiposity, participants selected 1 of 9 somatotype pictograms that best illustrated body shape and size at age 5 [27, 28]. Because few participants reported levels 5 and above, we collapsed these into a single category representing the greatest adiposity. Region of residence at age 15 was assessed in 1993. Childhood socioeconomic status, measured by parents’ educational attainment at the participant’s birth (high school or less, some college, 4+ years of college), was assessed in 2005. In the NHS II dataset, there is also information about participant SES. However, when assessing the model fit, participant SES showed a high correlation with parental education, and parental education explained the more substantial proportion of the association between TV viewing and PTSD. Hence, we selected parental education to represent overall SES. Physical activity was measured in 1991, 1997, 2001, and 2005 and categorized as <3, 3–8.9, 9–17.9, 18–26.9 and 27+ metabolic equivalent of task (MET)-hours/week. Physical activity in the NHS II has been validated using past-week activity and 7-day activity diaries over 1 year (r = 0.79) [29]. Physician diagnosis of depression was queried in 2003, 2005, 2007, and 2009. We included a “missing” category for all categorical covariates. For covariates other than region of residence, fewer than 5% of participants were missing data.

### Statistical analysis

We examined baseline characteristics in 1989 by concurrent trauma/PTSD symptoms. Next, we used linear mixed models to estimate the association between time-updated trauma/PTSD status and 18-year TV viewing trajectories. For these analyses, trauma/PTSD status was lagged by two years relative to TV viewing (e.g., PTSD status in 1990 predicted TV viewing in 1992) to reduce the likelihood that TV viewing measurements preceded (and might have altered) PTSD symptoms in a given year. We fit random intercept and random slope models, allowing individual differences in TV viewing at baseline as well as individual differences in change over time. Time was measured as the number of years from the first TV viewing measure to the end of follow-up and ranged from 0–18 years. Women with no trauma exposure were the reference group. In these analyses, the base Model 1 adjusted for age only, Model 2 further adjusted for race/ethnicity, region of residence, somatotype at age 5, parental education, and history of depression, and Model 3 added time-varying physical activity level.

Next, to examine TV viewing patterns prior to and following onset of PTSD, a random intercept random slope linear spline mixed model was fit to the subsample of women whose trauma exposure or onset of PTSD symptoms occurred during follow-up (i.e. between 1991 and 2009). For these analyses, women with trauma exposure but no PTSD symptoms served as the reference group. Time was measured as the number of years from trauma/PTSD symptom onset, ranging from -17 (17 years prior to onset) to +17 (17 years after onset) with zero (0) indicating the year of onset (which differed for each participant). For example, for women whose PTSD onset in 2000, the TV viewing measure in 2005 occurred at time = +5. If a woman had PTSD onset in 1998, TV watching in 1991 was coded as time = -7. Thus, there were 35 time points, from -17 to +17, including 0. We set the knot of the spline at time = 0, the point at which trauma/PTSD occurred, to facilitate examining separate patterns of TV viewing prior to and following trauma/PTSD onset.

Sensitivity analyses were conducted excluding participants who reported a life-threatening illness or serious car accident as their worst trauma because of potential confounding by physical disability.

## Results

Table 1 summarizes the distribution of covariates by trauma exposure and PTSD symptom categories at study baseline in 1989. At study inception, approximately half of the sample (n = 25,233) had experienced a traumatic event denoted as their worst one. By 2008, 19.9% of women had no history of trauma and 6.6% reported the most severe PTSD symptom level (6–7 symptoms). There was no significant difference in physical activity levels across trauma/PTSD groups. However, the proportion of women reporting lifetime depression increased with severity of PTSD symptoms; compared to 22% of women with no trauma, 60% of women with 6–7 PTSD symptoms reported prior lifetime depression.

**Table 1:** Sample characteristics at baseline (1989), stratified by number of symptoms of posttraumatic stress disorder (N = 50,020).

	No Trauma (n = 24,787, 49.6%)	Trauma, No Symptoms (n = 15,319, 30.6%)	Trauma, 1–3 Symptoms (n = 4,943, 9.9%)	Trauma, 4–5 Symptoms (n = 3,001 6.0%)	Trauma, 6–7 Symptoms (n = 1,978, 3.9%)
Age, yrs (SD)	34.28 (4.7)	35.21 (4.5)	35.34 (4.5)	35.22 (4.4)	35.09 (4.4)
TV Viewing, hrs/wk (SD)	8.83 (8.3)	8.53 (8.1)	8.56 (8.2)	8.45 (8.3)	8.55 (8.7)
Race, white (%)	23,262 (93.9)	14,305 (93.4)	4,655 (94.2)	4,846 (94.8)	1,863 (94.6)
Parental education					
≥ College (%)	5,815 (23.5)	3,327 (27.7)	1,126 (22.8)	667 (22.2)	489 (24.8)
Age 5 somatotype, highest (%)	1,619 (6.5)	1,080 (7.1)	326 (6.6)	227 (7.6)	167 (8.5)
Age 15 region of residence (%)					
Northeast (%)	2,416 (9.8)	1,651 (10.8)	607 (12.3)	355 (77.8)	267 (13.6)
Midwest (%)	8,980 (36.2)	5,333 (34.8)	1,755 (35.5)	1,048 (34.9)	632 (32.1)
West (%)	5,572 (22.5)	3,255 (21.3)	1,035 (20.9)	633 (21.1)	413 (21.0)
South (%)	2,746 (11.1)	1,832 (12.0)	599 (12.1)	398 (13.3)	269 (13.7)
Puerto Rico or Non-US (%)	418 (1.7)	262 (1.7)	74 (1.5)	45 (1.5)	27 (1.4)
Lifetime depression, 2009 (%)	5,507 (22.2)	2,856 (18.6)	1,466 (29.7)	1,384 (46.1)	1,186 (60.2)
Physical activity (MET hrs/wk) (SD)	27.36 (62.4)	27.21 (64.4)	26.40 (62.4)	25.32 (49.2)	27.06 (62.1)

Abbreviations: yrs = years; hrs/wk = hours/week

MET = metabolic equivalent task. One metabolic equivalent (MET) is defined as the amount of oxygen consumed while sitting at rest and is equal to 3.5 ml O_2_ per kg body weight x min.

### Trauma, PTSD symptoms, and TV viewing across time

**Fig 2:**
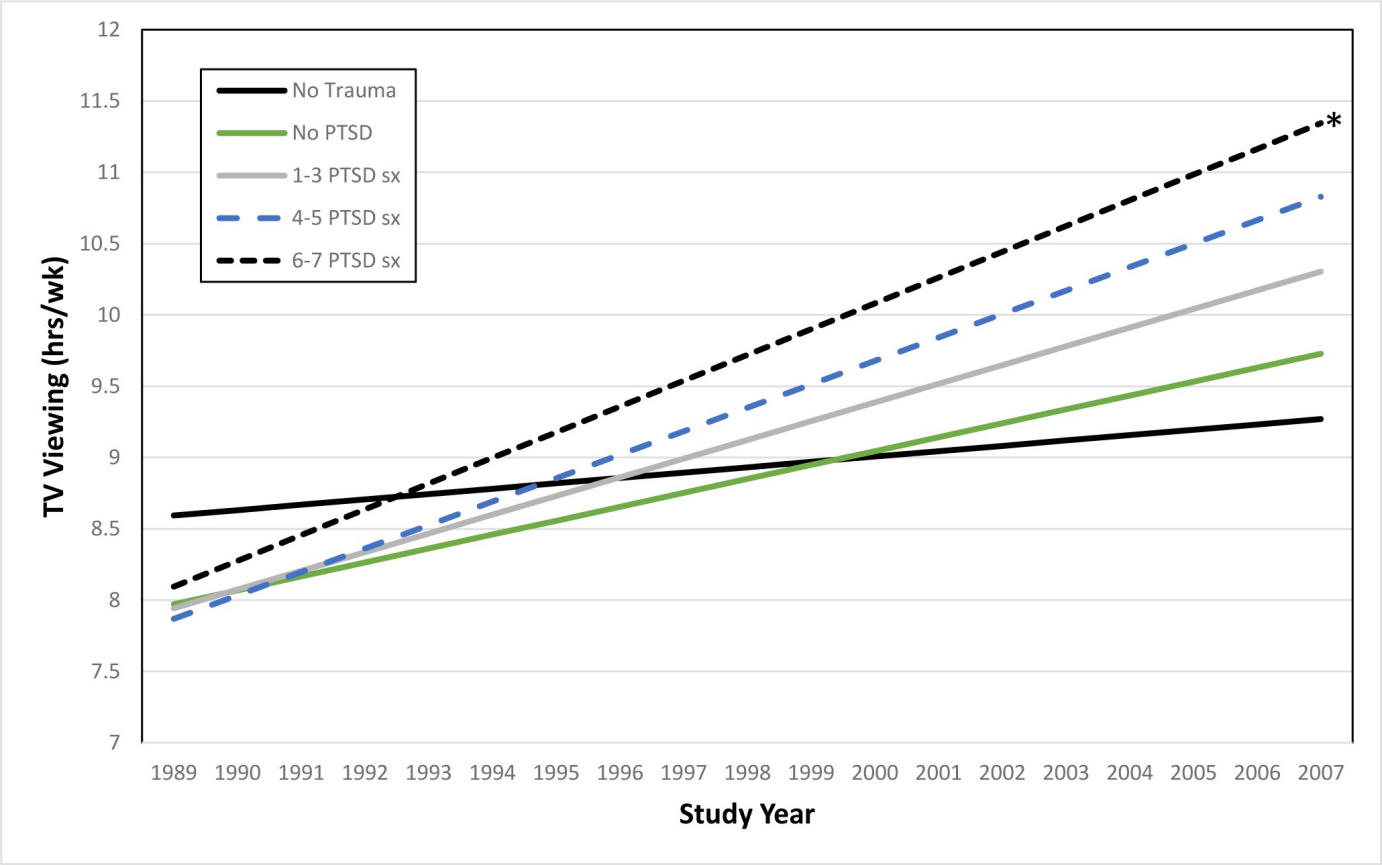
Age-adjusted predicted TV viewing trajectory over time by trauma/PTSD group among full sample (N = 50, 020) (*Groups with no PTSD(with Trauma exposure), 1–3 PTSD symptoms, 4–5 PTSD symptoms and 6–7 PTSD symptoms were all significantly different from the no trauma group, p<0.001).

The estimated TV viewing trajectories during the 18-year follow-up period by time-varying PTSD categories in the full sample (n = 50,020) are presented in Fig 2. Relative to women without trauma, TV viewing increased more steeply over time, in a dose-response fashion, among trauma exposed women who reported no PTSD symptoms (β = 0.06, SE = 0.01; p < .001), 1–3 symptoms (β = 0.09, SE = 0.01; p < .001), 4–5 symptoms (β = 0.13, SE = 0.01; p < .001) and 6–7 symptoms (β = 0.14, SE = 0.01, p < .001) in age-adjusted linear mixed models. Additional adjustment for race, parental education, region of residence, somatotype at age 5, and history of depression did not substantially change the results (Table A in S1 File). The difference in rate of increase in TV viewing between women with no trauma/no PTSD and women with 6–7 PTSD symptoms increased across follow-up, from the first TV measurement in 1991 (difference = 0.06 hours/week) to the last TV viewing measurement in 2009 (difference = 1.63 hours/week, Fig 2**)**.

**Fig 3:**
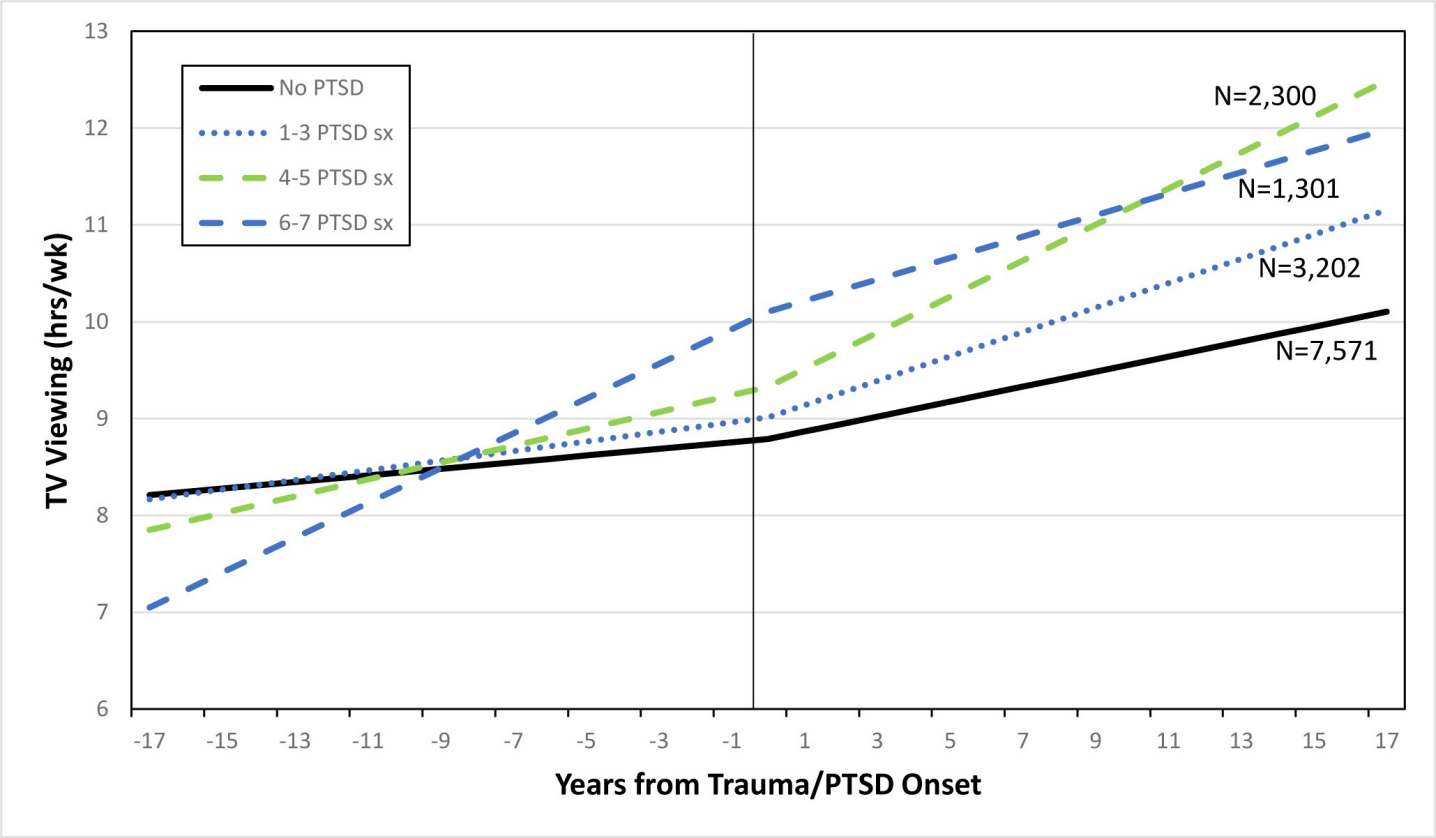
Age-adjusted predicted television viewing trajectory over time by trauma/PTSD group, before and after trauma/PTSD onset, among subsample. (n = 14, 374). Notes: Includes women with trauma/PTSD onset during follow-up, between 1991–2009 (n = 14, 374). Zero marks the year of trauma/PTSD onset. PTSD characterized in association with worst trauma.

Fig 3 shows the estimated TV viewing trajectories before and after the onset of trauma/PTSD symptoms among the subsample of women with trauma/PTSD during follow-up (N = 14,374). In the final model among trauma-exposed women, a steeper increase in TV viewing prior to trauma exposure was associated with subsequently developing more PTSD symptoms after trauma exposure (4–5 PTSD symptoms, β = 0.05, SE = 0.01, p = .002; 6–7 PTSD symptoms, β = 0.15, SE = 0.02, p < .001). Women reported few differences in the rate of change in TV viewing hours over time by PTSD category in the years following experiencing their worst trauma; women with 4–5 PTSD symptoms were the exception, reporting rates of TV viewing that increased more sharply than women with no PTSD (β = 0.11, SE = 0.03, p = .001). Results were similar in sensitivity analyses restricted to participants without a life-threatening illness or serious car accident (Tables B and C in S1 File).

## Discussion

This study examined the possibility of a bidirectional relationship between PTSD symptoms and TV viewing trajectories over time in women exposed to a wide range of traumatic events occurring in civilian settings. In longitudinal analyses, TV viewing increased more steeply over time in women with higher PTSD symptom levels in a dose-response fashion compared to women who were not trauma exposed. Although this difference was modest, it was relevant of a population level. Among the subsample of women who developed trauma/PTSD during follow-up, significantly steeper increasing in time spent viewing TV occurred *prior* to the onset of PTSD symptoms, compared to women who did not go on to develop PTSD symptoms. TV viewing continued to increase after PTSD onset among women with 4–5 PTSD symptoms, but not among women with 6–7 PTSD symptoms. Together, our findings suggest high rates of TV viewing may be a marker of vulnerability to developing PTSD in the event of trauma exposure; moreover these high levels of TV viewing persist after PTSD symptom onset, resulting in individuals with PTSD spending more time watching TV than their less distressed counterparts. Thus, PTSD onset may exacerbate tendencies to watch TV although whether PTSD causally contributes to this form of sedentary behavior remains to be determined. Importantly, all associations were independent of baseline demographic factors as well as depression and initial levels of physical activity.

Our results regarding time spent viewing TV extend findings from previous reports that focused on TV viewing of terrorist attacks and disasters. Those reports examined TV viewing as a potentially traumatic stressor in the context of pre-trauma TV viewing. Our data suggest that TV viewing more broadly is associated with an increased risk of developing PTSD [1–3, 30]. Regarding post-trauma TV viewing, we know of only one prior longitudinal study that has examined whether trauma onset results in a change in TV viewing [13]: the study indicated that participants watched more TV in the aftermath of the 9/11 attacks in the US. This study did not ascertain PTSD or other mental health symptoms.

Mechanisms linking TV viewing and PTSD are unknown. It is possible that women who are more socially isolated or have poorer coping skills are more likely to watch TV as well as develop PTSD when exposed to a traumatic stressor. However, once PTSD is established, it may also exacerbate TV viewing time. For example, women with PTSD may watch TV as a form of avoidance or numbing, which are hallmarks of PTSD. Women may also watch TV as a distraction from reminders of their traumatic event [31]. Persons with PTSD often showed diminished social involvement [8], and may chose TV viewing over social activities. We have previously shown in this cohort that women with PTSD engage in less physical activity compared with women without trauma exposure [11], and other studies have reported that PTSD patients identify a lack of motivation as a reason for ending their participation in sports (Lack of motivation: 24% before vs. 71% after PTSD onset)[32]. Women with PTSD may choose a passive activity, such as TV viewing, to occupy free time if their overall motivation to engage in other activities is low.

### Strengths and limitations

This study has numerous strengths, including longitudinal data, a large well-characterized study population, extensive data on potential confounding factors including data on trauma separate from PTSD symptoms, and repeated measures of TV viewing before and after the onset of PTSD symptoms.

This study has six important limitations. First, PTSD symptoms were only assessed once, retrospectively, in relation to the worst traumatic event. Although the date of PTSD onset was reported, it is possible that there was misclassification in timing and severity of PTSD symptoms. In particular, PTSD symptoms may have occurred before the worst event for women with multiple traumatic events[33]. Assessing PTSD in relation to the worst trauma is standard practice in studies that cannot assess PTSD related to all events due to time constraints[16]. However, these limitations in assessment will result in misclassification of some persons who have PTSD related to a trauma other than their worst as not having PTSD. Lifetime retrospective reporting usually leads to underestimation of lifetime prevalence, resulting in misclassification of cases as non-cases and likely biasing observed associations toward the null [34]. Also, measurement of TV viewing and PTSD symptoms were made in every two years simultaneously, which might cause some cases challenging to make subtle distinctions between pre- and post-trauma TV viewing. Second, from 1991 to 2009 many technological changes occurred, including the development of mobile phones and streaming media. These developments might result in increased hours of TV viewing overall, however, we could not measure other types of media watching on total hours of TV viewing. Third, information regarding the context in which the participant was watching TV, such as watching alone or with other people as a social activity, was not collected in this dataset. These conditions may represent different coping strategies, and have a differential influence on trauma response, which television viewing by oneself associated with isolation and more frequent TV viewing[35]. Unfortunately, study data did not allow us to distinguish the impact of the different TV viewing contexts on trauma response. Fourth, our findings may have limited generalizability, given our population of predominately white and well-educated professional female nurses from a specific age cohort. Fifth, although the original cohort was comprised of working nurses, change in their working status might have affected the results; women could have watched TV more after their retirement or upon ill health. Finally, the cohort began in 1989; however, PTSD was not assessed until 2008. The women in this study who reported PTSD symptoms may, therefore, represent healthy survivors. Thus, it is likely that our results represent conservative estimates of the true effect of PTSD on TV viewing.

### Implications/Conclusions

The current study shows that excessive TV viewing is a marker for increased vulnerability to PTSD; this finding parallels associations between TV viewing and other adverse mental health outcomes including depression [9], antisocial behavior, and ADHD [10]. We hypothesize that TV viewing is not a causal risk factor, but rather is a marker for limited social or community involvement, or a more avoidant coping style, which increases risk of developing PTSD subsequent to trauma. With regard to increased TV viewing after PTSD onset, there is rigorous evidence of increased cardiometabolic disease risk among persons with PTSD [23, 36, 37] and increased TV viewing hours may be a mechanism that links PTSD with cardiometabolic disease.

This study suggests some important considerations for clinicians as well as future directions for research. If future work replicates the finding of higher TV viewing among individuals suffering from PTSD, this may point to potential interventions that could reduce risk of secondary health outcomes. Interventions targeting lifestyle factors in people with PTSD, such as decreasing TV viewing and increasing physical activity, may help prevent cardiometabolic disease. However, current clinical management of PTSD patients rarely covers lifestyle modifications; moreover, clinicians are not focused on physical sequelae of PTSD, including cardiovascular diseases and diabetes. Lifestyle modification including reduction of sedentary behaviors, such as TV viewing, is feasible in this patient population; this is supported by results of a recent meta-analysis of physical activity among PTSD patients, which suggested that physical activity was more effective at decreasing PTSD symptoms among people with PTSD compared to controls [38]. Moreover, it may be helpful to design future work to test explicitly whether excessive TV viewing reliably serves as an early indicator that individuals are susceptible to PTSD (or other mental health problems), and identifying the specific factors related to TV viewing that particularly increase susceptibility. Additionally, it will be important to establish how common this phenomenon is, since if it is widespread, it could inform development of prevention strategies. For example, if in fact, these individuals are more likely to be on other screens (i.e., computer, cell phones, ipads), findings on TV viewing or other screen time may suggest the possibility that digital phenotyping may be particularly informative [39]. Further work in this area may provide new insights into early warning signs for susceptibility as well as interventions that might be effective for reducing risk of poor health outcomes among individuals who have or are at high risk of PTSD.

## Supporting information

S1 File (Table A) Linear mixed models assessing change in TV viewing (hrs/wk) between 1991–2009, by time-updated trauma exposure and/or PTSD symptoms with 2 years lagging effect (n = 50,020). (Table B) Linear mixed models assessing change in TV viewing (hrs/wk), 1991–2009, by time-updated trauma exposure and/or PTSD symptoms with 2 years lagging effect excluding individuals reporting worst trauma due to life-threatening medical illness or physical injury from car accidents. (n = 40,193). (Table C) Linear spline mixed models assessing change in TV viewing (hrs/wk), 1991–2009, before and after onset of trauma exposure and/or PTSD symptoms among women whose PTSD onset during study follow-up, excluding individuals reporting worst trauma due to life-threatening medical illness or from physical injury by car accident with 2 years lagging effect (n = 9,727).
(DOCX)
