# Supplementary material for: Posttraumatic stress disorder symptoms and television viewing patterns in the Nurses’ Health Study II: A longitudinal analysis
Source: PLoS One. 2019 Mar 21;14(3):e0213441. doi: 10.1371/journal.pone.0213441 (PMC6428392; doi:10.1371/journal.pone.0213441)
Supplement: S1 File — (Table A) Linear mixed models assessing change in TV viewing (hrs/wk) between 1991–2009, by time-updated trauma exposure and/or PTSD symptoms with 2 years lagging effect (n = 50,020). (Table B) Linear mixed models assessing change in TV viewing (hrs/wk), 1991–2009, by time-updated trauma exposure and/or PTSD symptoms with 2 years lagging effect excluding individuals reporting worst trauma due to life-threatening medical illness or physical injury from car accidents. (n = 40,193). (Table C) Linear spline mixed models assessing change in TV viewing (hrs/wk), 1991–2009, before and after onset of trauma exposure and/or PTSD symptoms among women whose PTSD onset during study follow-up, excluding individuals reporting worst trauma due to life-threatening medical illness or from physical injury by car accident with 2 years lagging effect (n = 9,727). (DOCX) [file pone.0213441.s001.docx]

(Table A) Linear mixed models assessing change in TV viewing (hrs/wk) between 1991-2009, by time-updated trauma exposure and/or PTSD symptoms with 2 years lagging effect (n=50,020).

|  | Model 1 ^a^ | | Model 2 ^b^ | | Model 3 ^c^ | |
| --- | --- | --- | --- | --- | --- | --- |
|  | b Value (SE) | P Value | b Value (SE) | P Value | b Value (SE) | P Value |
| No Trauma | Ref | Ref | Ref | Ref | Ref | Ref |
| 0 PTSD Sx | 0.060 (0.006) | <0.001 | 0.059 (0.006) | <0.001 | 0.059 (0.006) | <0.001 |
| 1-3 PTSD Sx | 0.094 (0.009) | <0.001 | 0.094 (0.009) | <0.001 | 0.093 (0.009) | <0.001 |
| 4-5 PTSD Sx | 0.127 (0.011) | <0.001 | 0.129 (0.011) | <0.001 | 0.129 (0.011) | <0.001 |
| 6-7 PTSD Sx | 0.143 (0.013) | <0.001 | 0.147 (0.013) | <0.001 | 0.146 (0.013) | <0.001 |

a Adjusts for age at baseline

b Adjusts for age, race, parental education, region at age 15, highest age 5 somatotype, and history of depression

c Adjusts for age, race, parental education, region at age 15, highest age 5 somatotype, history of depression, and physical activity

(Table B) Linear mixed models assessing change in TV viewing (hrs/wk), 1991-2009, by time-updated trauma exposure and/or PTSD symptoms with 2 years lagging effect excluding individuals reporting worst trauma due to life-threatening medical illness or physical injury from car accidents. (n=40,193).

|  | Model 1 ^a^ | | Model 2 ^b^ | | Model 3 ^c^ | |
| --- | --- | --- | --- | --- | --- | --- |
|  | β Value (SE) | P Value | β Value (SE) | P Value | β Value (SE) | P Value |
| No Trauma | Ref | Ref | Ref | Ref | Ref | Ref |
| 0 PTSD symptoms | 0.045 (0.007) | <0.001 | 0.044 (0.007) | <0.001 | 0.044 (0.007) | <0.001 |
| 1-3 PTSD symptoms | 0.085 (0.010) | <0.001 | 0.085 (0.010) | <0.001 | 0.085 (0.010) | <0.001 |
| 4-5 PTSD symptoms | 0.095 (0.012) | <0.001 | 0.098 (0.012) | <0.001 | 0.097 (0.012) | <0.001 |
| 6-7 PTSD symptoms | 0.113 (0.015) | <0.001 | 0.117 (0.015) | <0.001 | 0.116 (0.015) | <0.001 |

a Adjusts for age at baseline

b Adjusts for age, race, parental education, region at age 15, highest age 5 somatotype, and history of depression

c Adjusts for age, race, parental education, region at age 15, highest age 5 somatotype, history of depression, and physical activity

(Table C) Linear spline mixed models assessing change in TV viewing (hrs/wk), 1991-2009, before and after onset of trauma exposure and/or PTSD symptoms among women whose PTSD onset during study follow-up, excluding individuals reporting worst trauma due to life-threatening medical illness or from physical injury by car accident with 2 years lagging effect (n=9,727).

|  | Model 1 ^a^ | | Model 2 ^b^ | | Model 3 ^c^ | |
| --- | --- | --- | --- | --- | --- | --- |
|  | b Value (SE) | P Value | b Value (SE) | P Value | b Value (SE) | P Value |
| Before PTSD onset | | | | | | |
| 1-3 PTSD Sx | 0.028 (0.020) | 0.154 | 0.027 (0.020) | 0.187 | 0.029 (0.020) | 0.171 |
| 4-5 PTSD Sx | 0.055 (0.022) | 0.008 | 0.055 (0.013) | 0.013 | 0.058 (0.022) | 0.014 |
| 6-7 PTSD Sx | 0.126 (0.028) | <0.001 | 0.128 (0.029) | <0.001 | 0.127 (0.028) | <0.001 |
| After PTSD onset | | | | | | |
| 1-3 PTSD Sx | 0.007 (0.043) | 0.868 | 0.008 (0.043) | 0.857 | 0.007 (0.043) | 0.878 |
| 4-5 PTSD Sx | 0.022 (0.049) | 0.653 | 0.022 (0.049) | 0.653 | 0.023 (0.049) | 0.642 |
| 6-7 PTSD Sx | -0.209 (0.063) | 0.001 | -0.209 (0.063) | <0.001 | -0.210 (0.063) | <0.001 |

a Adjusts for age at baseline

b Adjusts for age, race, parental education, region at age 15, highest age 5 somatotype, and history of depression

c Adjusts for age, race, parental education, region at age 15, highest age 5 somatotype, history of depression, and physical activity
